# Impact of the COVID-19 pandemic in a substance use harm reduction setting

**DOI:** 10.1192/j.eurpsy.2021.2184

**Published:** 2021-08-13

**Authors:** P. Frias, J. Vilares

**Affiliations:** 1 Psychiatry, Hospital magalhães Lemos, Porto, Portugal; 2 Giru Gaia, APDES, Gaia, Portugal

**Keywords:** Substance use disorders, COVID-19, Harm Reduction, Community-based Mental Healthcare

## Abstract

**Introduction:**

Harm reduction (HR) approaches substance use disorders (SUDs) from a standpoint of humanism and tolerance, understanding the difficulty of terminating consumption in some cases, and instead promoting practices aimed at reducing risks inherent to substance use while granting accessibility to comunity based healthcare and contact with differentiated care, when needed, to patients who otherwise wouldn’t have access to it. The COVID-19 pandemic brought about an economic crises, impacting mainly people from lower classes, associated with an increased prevalence of heroine and crack cocaine use and exacerbating previous SUDs.

**Objectives:**

Describe changes in a HR population treated in HR context in Gaia, Portugal.

**Methods:**

Data was retrieved from clinical files of patients undergoing treatment in a Harm Reduction setting on the HR team “GiruGaia” from Porto. Analysed data included number of patients admitted to treatment in the period between march the 1st and december the 30th 2020, their sociodemographic data, psychiatric and substance use history, psychiatric treatment and opioid agonist treatment when required. Gathered data was compared to the same time period in previous years.

**Results:**

The number of patients admitted to treatment drastically increased. Patients presented with poorer socioeconomic conditions, more psychiatric comorbidity and in need of more extensive treatment interventions.

**Conclusions:**

Our findings suggest that the pandemic and it’s socioeconomic impact affected drug users in need of HR interventions worsening underlying psychiatric disorders, poverty and inacessibility to healthcare. Drug users are often a forgotten population, and our results indicate that more attention should be devoted to them.

**Disclosure:**

No significant relationships.

